# High-Intensity Interval Training with Vibration as Rest Intervals Attenuates Fiber Atrophy and Prevents Decreases in Anaerobic Performance

**DOI:** 10.1371/journal.pone.0116764

**Published:** 2015-02-13

**Authors:** Sandro Manuel Mueller, David Aguayo, Matthias Zuercher, Oliver Fleischmann, Urs Boutellier, Maria Auer, Hans H. Jung, Marco Toigo

**Affiliations:** 1 Exercise Physiology Lab, Institute of Human Movement Sciences, ETH Zurich, Zurich, Switzerland; 2 Department of Neurology, University Hospital Zurich, Zurich, Switzerland; Universidad Pablo de Olavide, Centro Andaluz de Biología del Desarrollo-CSIC, SPAIN

## Abstract

Aerobic high-intensity interval training (HIT) improves cardiovascular capacity but may reduce the finite work capacity above critical power (W′) and lead to atrophy of myosin heavy chain (MyHC)-2 fibers. Since whole-body vibration may enhance indices of anaerobic performance, we examined whether side-alternating whole-body vibration as a replacement for the active rest intervals during a 4x4 min HIT prevents decreases in anaerobic performance and capacity without compromising gains in aerobic function. Thirty-three young recreationally active men were randomly assigned to conduct either conventional 4x4 min HIT, HIT with 3 min of WBV at 18 Hz (HIT+VIB18) or 30 Hz (HIT+VIB30) in lieu of conventional rest intervals, or WBV at 30 Hz (VIB30). Pre and post training, critical power (CP), W′, cellular muscle characteristics, as well as cardiovascular and neuromuscular variables were determined. W′ (−14.3%, P = 0.013), maximal voluntary torque (−8.6%, P = 0.001), rate of force development (−10.5%, P = 0.018), maximal jumping power (−6.3%, P = 0.007) and cross-sectional areas of MyHC-2A fibers (−6.4%, P = 0.044) were reduced only after conventional HIT. CP, V̇O2peak, peak cardiac output, and overall capillary-to-fiber ratio were increased after HIT, HIT+VIB18, and HIT+VIB30 without differences between groups. HIT-specific reductions in anaerobic performance and capacity were prevented by replacing active rest intervals with side-alternating whole-body vibration, notably without compromising aerobic adaptations. Therefore, competitive cyclists (and potentially other endurance-oriented athletes) may benefit from replacing the active rest intervals during aerobic HIT with side-alternating whole-body vibration.

**Trial Registration:**

ClinicalTrials.gov Identifier: NCT01875146

## Introduction

High-intensity interval training (HIT) consists of a variety of high-intensity exercises interspersed with rest intervals. It is known that HIT is a very efficient way to improve cardiovascular variables [1] and aerobic function, *e*.*g*. critical power (CP), leading to improved endurance performance [2–3]. Because of the considerable training effect, HIT is used in a variety of sports, encompassing a wide range from classical endurance sports to team sports. Accordingly, numerous HIT protocols have been established to meet the requirements associated with specific sport types. Because of this, HIT protocols usually differ in regards to intensity (*e*.*g*. in terms of % peak power), duration and specific sequence of the exercise intervals. With respect to cycling, it was shown that 4 min high-intensity intervals interspersed with 1.5 min low-intensity intervals lead to the highest increase in 40 km cycling time trial speed and peak power, as compared to shorter high-intensity intervals [4]. The highest peak heart rate and peak oxygen uptake (*V̇*O_2peak_) measurements during running-based HIT were attained with a 1:1 work to rest ratio by using 4 min intervals as compared to 1, 2 and 6 min intervals [5]. As the attainment and sustaining of high heart rates and high *V̇*O_2_-values during HIT seem to be a prerequisite for cardiovascular adaptations [6], 4 min high-intensity intervals with 4 min rest intervals may be recommended in order to achieve optimal training effects. In this regard, it was found that a 4x4 min HIT, which includes 3 min of low-intensity intervals increases *V̇*O_2peak_ and stroke volume to a higher extent than long slow distance running or running at the lactate threshold [7]. These authors also stated that from a practical point of view a 4x4 min HIT was easier to administer than HIT with shorter high-intensity intervals and shorter rest intervals [7]. Finally, irrespective of the interval lengths, HIT leads to a more oxidative metabolism [8] and to a reduction in proportion of myosin heavy chain (MyHC)-2X fibers [9]. These adaptations might further increase fatigue resistance and thus benefiting endurance performance.

Despite the proven benefits, there appear to be some pitfalls in using HIT. For example, the shift to a more oxidative metabolism and the alteration in MyHC fiber type distribution might reduce anaerobic performance and capacity. It was shown that countermovement and squat jump power were reduced after HIT [10], and that cross-sectional area (CSA) of MyHC-2 fibers tended to be reduced after HIT [11]. Notably, a reduced share of MyHC-2 fibers is associated with a lower maximal muscular shortening velocity [12]. For a given physiological muscle CSA with given fiber type area distribution, the reduction in maximal shortening velocity will lead to a reduction in maximum muscle power. Further evidence for a decrease in anaerobic capacity is that the finite work capacity above CP (*W′*) tended to decrease after only 4 weeks of HIT in favor of the increase in CP, as shown by the significant negative correlation between ∆CP and ∆*W′* [3]. Notably, these studies have shown that both the decrease in *W′* and maximum muscle power prove to be disadvantageous in most endurance-oriented sports (*e*.*g*. hill climbing, sprinting), despite the observed gain in endurance performance achieved by applying HIT.

To avoid the decreases in *W′* and maximum muscle power that might come with HIT, the option of adjusting the rest interval between the high-intensity bouts has been proposed [13–14]. However, the sole modification of cycling exercise intensity during the rest intervals may not attenuate the decrease in anaerobic performance without compromising the gain in aerobic performance. In addition, studies that investigated the effects of the rest intervals on training adaptations [13–14] were aimed primarily at increasing aerobic adaptations or at making training more bearable, while attaining an identical training effect. To our knowledge, there have been no studies presented that investigated whether the modification of rest intervals might prevent the afore-mentioned decreases in anaerobic performance and capacity. Whole-body vibration (WBV) is a training stimulus that might attenuate the aforementioned anaerobic loss. Despite the scientific controversy on the possible training effects of WBV, which is based on contradictory results, WBV training has been shown to increase several markers of anaerobic performance: leg press power [15], jumping height [16], one-repetition maximum [17], and Wingate test mean power [18]. In addition, improvements in knee extension power were paralleled by increased muscle mass [19]. WBV might have the potential to reduce HIT-specific decreases in anaerobic performance and/or capacity. An additional appealing aspect that might be relevant to HIT is the reduced pain sensation associated with WBV [20]. In fact, HIT is associated with high ratings of subjectively perceived exertion [21]. This factor presents special challenges involving attitudes surrounding HIT usage. If WBV performed during the rest intervals significantly reduces leg pain sensation during the high-intensity cycling intervals, the implementation of these high-intensity intervals could be facilitated, by reducing negative perceptions that may lead to motivational deficits. Further, it must be pointed out that in most applied HIT-protocols, the warm-up, rest intervals, and cool-down phases account for more than 50% of total exercise time. As the rest intervals are as time-consuming as the high-intensity intervals, it would prove advantageous to increase effectiveness of these phases.

For a given vibration type (side-alternating vertical sinusoidal vibration *vs*. vertical synchronous vibration) and amplitude, acute effects of WBV are dependent on vibration frequency. With respect to side-alternating vertical sinusoidal vibration, low frequencies (<20 Hz) allow complete tension-relaxation cycles, based on an average duration of a complete tension-relaxation cycle of ~50 ms. Hence, frequencies above 20 Hz consist of stimuli that do not allow complete muscle relaxation to occur. Accordingly, it is known that higher vibration frequencies lead to higher relative *V̇*O_2_ [22] and that with frequencies from 5 to 30 Hz, electromyography activities increase in some (but not all) muscles of the lower body [23]. In particular, electromyography activity is similar in the thigh muscles, where vibration frequencies >15 Hz are applied [23]. Furthermore, based on our experience, side-alternating WBV at 18 Hz is less demanding, and is associated with a lower rating of perceived exertion than WBV at 30 Hz. Provided that the similar acute and training effects with WBV at 18 and 30 Hz are achieved, the lower vibration frequency might thereby facilitate execution of HIT. Currently, however, little is known about the differences in training adaptations as well as acute physiological variables (*e*.*g*. pain sensation) with the use of different vibration frequencies.

In our current study, we investigated whether the replacement of the active rest intervals during a 4x4 min HIT with side-alternating WBV could prevent the decrease in anaerobic capacity and performance, specifically *W′*, peak knee extensor torque, maximum jumping power and maximum rate of force development (RFD), without compromising the gains in CP and *V̇*O_2peak_. In addition, we posed the question as to whether the side-alternating WBV intervals would decrease the rating of perceived exertion during the high-intensity cycling intervals. Based on the reported training adaptations following WBV and acute effects of different rest interval intensities, we hypothesized that the combination of HIT and side-alternating WBV leads to similar adaptations in CP and cardiovascular variables, as compared to the original 4x4 min HIT-protocol [7], but at the same time, impedes reductions in *W′*, peak knee extensor torque, maximum jumping power and RFD. Additionally, based on the similar electromyography activity of the thigh muscles with vibration frequencies >15 Hz, we hypothesized that 18 and 30 Hz lead to similar training adaptations, despite the difference in the tension-relaxation cycle. Training adaptations for the combination between HIT and side-alternating WBV were controlled by including a group performing side-alternating WBV only, using the same duty cycle as for the HIT groups.

## Methods

### Participants

Thirty-six recreationally active males volunteered to participate in this study. The participants were recruited by placard. They were assigned to one of four training groups matched for CP∙kg^−1^ lean mass: 1) Conventional 4x4 min cycling HIT (HIT), 2) HIT with WBV at 18 Hz being applied in lieu of conventional rest intervals (HIT+VIB18), 3) HIT with WBV at 30 Hz instead of conventional rest intervals (HIT+VIB30), and 4) only WBV at 30 Hz (VIB30) with the same duty cycle as used for the conventional rest intervals. Prior to this study, the participants were instructed to maintain their individual training routines in terms of training frequency and training intensity, and were advised not to include new or additional high-intensity exercise during the study period. Three participants withdrew from the study due to personal reasons not related to the trial. This led to the following characteristics for the HIT (*n* = 8, 26.0 ± 5.2 years, 180.0 ± 4.2 cm, 79.2 ± 11.2 kg, 49.2 ± 8.2 ml∙min^−1^∙kg^−1^ body mass *V̇*O_2peak_), HIT+VIB18 (*n* = 8, 27.6 ± 3.3 years, 181.2 ± 5.9 cm, 79.3 ± 7.0 kg, 48.4 ± 5.1 ml∙min^−1^∙kg^−1^ body mass *V̇*O_2peak_), HIT+VIB30 (*n* = 9, 27.5 ± 4.5 years, 180.6 ± 8.1 cm, 81.8 ± 10.2 kg, 48.9 ± 6.1 ml∙min^−1^∙kg^−1^ body mass *V̇*O_2peak_), and VIB30 (*n* = 8, 27.8 ± 3.6 years, 179.0 ± 9.7 cm, 79.9 ± 11.5 kg, 48.1 ± 4.9 ml∙min^−1^∙kg^−1^ body mass *V̇*O_2peak_) groups. There were no statistically significant differences in physical and performance characteristics among the groups prior to the training period. Participants were fully informed about the purposes, benefits and risks associated with the study, and completed a routine health questionnaire before giving written informed consent to participate in the study. The study was approved by the ethics committee of the canton Zurich, and was conducted in accordance with the declaration of Helsinki.

### Training regimen

The participants reported alternately two and three times per week to the laboratory for the supervised training sessions. Each participant completed thereby twenty training sessions during the 8-week training phase. Prior to the three HIT regimens, the participants completed a warm-up of 3 min at 40% peak power (*P*_peak_). HIT consisted of four high-intensity intervals at 75% *P*_peak_, alternating with active rest intervals of 4 min duration at 40% *P*_peak_ (adapted from Helgerud *et al*. [7]) on a cycle ergometer (Bike XT, Technogym, Gambettola, Italy). The additional minute of rest interval was implemented to adjust for the transitions from cycle ergometer to vibration plate and *vice versa* in the HIT+VIB groups. Hence, the time requirement to switch between the cycle ergometer and the vibration plate (and *vice versa*) was 30 s. In the HIT+VIB18 and HIT+VIB30 groups, the low-intensity cycling intervals were replaced by standing still with feet shoulder-width apart in a half-squat position on a side-alternating Galileo vibration plate (Novotec, Pforzheim, Germany), oscillating at 18 (HIT+VIB18) or 30 Hz (HIT+VIB30) for 3 min. Sole WBV training (VIB30) was executed identically as the rest intervals in the groups combining HIT and WBV at a vibration frequency of 30 Hz. The vibration amplitudes (displacement from baseline to peak) were 2.98 ± 0.49, 2.97 ± 0.54, and 3.56 ± 0.49 mm for the HIT+VIB18, HIT+VIB30, and VIB30 groups, respectively. Amplitudes did not differ significantly between the training groups. All WBV participants wore non-slippery socks to avoid any dampening of shoe soles during vibration training. To assure identical fixation of the feet during cycling, the pedals of the ergometer allowed strapping of the feet with shoes (HIT) as well as with non-slippery socks (HIT+VIB18 and HIT+VIB30). Every participant always completed four 4 min high-intensity intervals. It occurred that power output had to be reduced at the very end of a high-intensity interval in order to just fulfill the 4-min requirement. Power output was only increased from one training session to the next if the participant had successfully completed all four high-intensity intervals at the predetermined power output. As soon as an individual participant of the HIT, HIT+VIB18, and HIT+VIB30 groups was able to sustain all 4 high-intensity intervals successfully at the predetermined level without reducing the power output at the end of an interval, the power of the warm-up, high-intensity intervals and rest intervals were increased by 3% *P*_peak_ for the upcoming training session.

### Experimental procedures

The study consisted of pre- and post-tests for all participants and 8 weeks of either HIT (HIT, HIT+VIB18, and HIT+VIB30) or WBV training alone (VIB30). On the first testing day, a percutaneous muscle biopsy was obtained from the *M*. *vastus lateralis* using a 6-mm Bergström needle (Dixons Surgical Instruments, Essex, UK) as previously described [24]. On the second testing day, participants completed an incremental ramp cycle ergometer test to determine *P*_peak_, *V̇*O_2peak_, peak cardiac output, and peak stroke volume. On that occasion, seat and handlebar of the cycle ergometer (Ergoselect 200 K, Ergoline, Bitz, Germany) were adjusted. These settings were adopted for usage in all of the consecutive trials. After a 3 min resting measurement, the ramp test started at 100 W and involved power increases of 9 W every 18 s (30 W∙min^−1^) until volitional exhaustion set in. The participants were free to choose the cadence, but the initially chosen revolutions per minute (rpm) had to be maintained throughout all the following cycling tests. Volitional exhaustion, *i*.*e*. task failure, for all cycling tests was defined as the point in time when participants stopped pedaling or the cadence fell more than 5 rpm for > 5 s. In further testing sessions with at least 24 h of rest in between, a total of four constant-load cycle ergometer tests were completed in a randomized order to determine CP [25], as previously described [26]. Briefly, after a warm-up of 5 min at 75 W the constant-load tests were conducted at 85, 90, 95, and 105% *P*_peak_. These constant-load tests resulted in exhaustion between 99 and 572 s. We then calculated CP and the finite work capacity above CP (*W′)* by applying the linear power-time^−1^ equation:

$$power=CP+W^{\prime }•time^{-1}$$

The coefficient of determination (*R* ^2^) for CP pre- and post-training was 0.99 ± 0.01 and 0.99 ± 0.01 for the HIT group, 0.99 ± 0.01 and 0.99 ± 0.02 for the HIT+VIB18 group, 0.99 ± 0.01 and 0.99 ± 0.01 for the HIT+VIB30 group, as well as 0.99 ± 0.01 and 0.99 ± 0.01 for the VIB30 group, respectively. The standard error of estimate (SEE) for CP pre- and post-training was 3.60 ± 1.46 W and 3.96 ± 2.39 W for the HIT group, 3.26 ± 1.06 W and 3.86 ± 2.91 W for the HIT+VIB18 group, 3.98 ± 2.53 W and 3.62 ± 2.31 W for the HIT+VIB30 group, as well as 3.19 ± 1.17 W and 3.37 ± 1.78 W for the VIB30 group, respectively. The typical error, expressed as a coefficient of variation, determined in our laboratory for CP, *V̇*O_2peak_, *P*_peak_, peak cardiac output, peak heart rate, and peak stroke volume, was 3.8%, 3.7%, 2.3%, 2.0%, 1.8%, and 2.3%, respectively. Prior to the third constant-load test, participants underwent jumping mechanography measurements, while knee extension dynamometry measurements were conducted prior to the last constant-load test of the pre-test session. During the post testing, all exercise tests were performed in an identical order.

### Equipment and measurements

*Cycling tests*. During all cycling tests and the first and twentieth training sessions, participants were equipped with a facemask, which covered their mouth and nose (Hans Rudolph, Shawnee, KS, USA). The facemask was connected with an anti-bacterial filter (PALL PRO1087, Pall, East Hills, NY, USA) to an Innocor device (Innocor, Innovision, Odense, Denmark). Pulmonary gas exchange and ventilation were continuously measured breath by breath during the ergometer trials. *V̇*O_2peak_ was determined as the highest mean of *V̇*O_2_-values over a 10-s period during the incremental ramp cycle ergometer test. Heart rate was continuously recorded during all cycling tests and all training sessions (S610i, Polar Electro, Kempele, Finland). Peak cardiac output was determined during maximal exercise of the incremental ramp cycling test using an Innocor inert gas rebreathing unit (Innocor, Innovision, Odense, Denmark). Peak stroke volume was calculated by dividing peak cardiac output by the corresponding heart rate.

**Acute effects during the first and twentieth training sessions.** At the end of each high-intensity interval, participants were asked to rate their individual perception of breathlessness, respiratory exertion, leg exertion, and pain on a visual analog scale. The scale consisted of a horizontal line with the word “none” at the left end of the scale, and “maximal” at the right end of the scale. For quantification, each completed visual analog scale was retrospectively scored from 0 to 10. During the last 30s of each interval, a 20µl sample of arterialized venous blood was taken from the earlobe for the measurement of blood lactate concentration. Blood samples were analyzed with a BIOSEN C_line Sport (EKF-diagnostic, Barleben, Germany).

**Knee extension.** Knee extensor maximal voluntary torque (MVT) and rate of force development (RFD) were tested using a dynamometer (Con-Trex MJ, Physiomed Elektromedizin, Schnaittach/Laipersdorf, Germany) sampling at 512 Hz. The body of each participant was stabilized with straps and handles. Afterwards, the participants performed 3 maximal knee extensions (*ω* = 3.14 rad∙s^−1^) that were separated by 1 min, for the purpose of assessing MVT. Subsequently, the lever arm of the dynamometer was fixed at a knee angle of 110° (full extension = 180°) and the participants were advised to extend their leg as fast and as powerful as possible. RFD was calculated for the time interval between 0 and 200 ms. Onset of torque production was defined as baseline +7.5 N∙m [27]. The attempt with the highest MVT and RFD, respectively, was used for analysis. The typical error, expressed as a coefficient of variation, determined in our laboratory for MVT and RFD was 4.7% and 10.1%, respectively.

**Jumping mechanography.** Three vertical countermovement jumps (CMJ) with freely moving arms (separated by 30 s of rest) were performed on a Leonardo Mechanograph force plate (Novotec Medical, Pforzheim, Germany) in order to determine maximal jumping power (CMJ *P*_max_) and peak velocity. Data detection, storage, and analysis were performed with the manufacturer’s software (Leonardo Mechanography GRFD version 4.4, Novotec, Pforzheim, Germany). The CMJ with the highest jumping height was used for analysis. The typical error expressed as a coefficient of variation, determined in our laboratory for CMJ *P*_max_ was 3.7%.

### Muscle biopsy analysis

**Figure 1:**
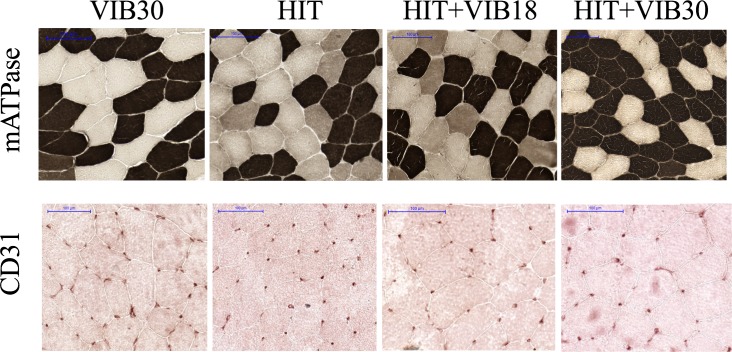
Representative images for mATPase and CD31 stainings in the whole-body vibration (VIB30), high-intensity interval training (HIT), and combined HIT and whole-body vibration at 18 Hz (HIT+VIB18) as well as at 30 Hz (HIT+VIB30) groups. Black fibers, myosin heavy chain (MyHC)-1; grey fibers, MyHC-2A; intermediate fibers, MyHC-2X; brown dots, capillaries.

Tissue sections were cut at 10-*μ*m thickness in a cryostat (CM3050 S, Leica, Wetzlar, Germany) maintained at −25°C, and mounted on Fisherbrand Superfrost/Plus microscope slides (Fisher Scientific, Pittsburgh, PA, USA). The serial cryocut cross-sections were stained using the myofibrillar adenosintriphosphatase (mATPase, Fig. 1) method at pH 4.6 as previously described [28]. On average, 707 ± 254 muscle fibers per participant were classified, according to their myosin heavy chain (MyHC) isoform into MyHC-1, MyHC-2A and MyHC-2X. As marker for muscle capillaries, the monoclonal mouse anti-human CD31 endothelial antibody (DAKO, Carpinteria, Canada, 1:600) was used (Fig. 1). Overall capillary-to-fiber ratio was calculated by dividing the number of CD31-positive cells by the number of muscle fibers. On average 253 ± 110 capillaries per participant were counted for the analysis of the capillary-to-fiber ratio. We used the NIH Image J Software (version 1.46r, National Institutes of Health, Bethesda, MD, USA) for the fiber and capillary counts. Fiber CSA was determined by fully encircling the borders of the mATPase stained cells with Adobe Photoshop Pro CS6 (Adobe Systems, San Jose, CA, USA) of at least 50 fibers per MyHC isoform [29]. Fiber circularity was calculated using the formula (4π∙*CSA*)/(*perimeter*)^2^, and only fibers with a circularity higher than 0.7 were considered for analysis (perfect circle = 1.0). Because of the non-occurrence of MyHC-2X fibers in some individuals, CSA data are solely presented for MyHC-1 and MyHC-2A.

### Statistical analysis

Data are presented as mean values ± standard deviations (SD). Normality of data was visually ascertained by Q-Q-plots. Pre-test values between the training groups as well as acute training values were analyzed with a one-way analysis of variance. For the detection of significant differences between groups over time, a univariate general linear model was applied. For this analysis, the differences post−pre (∆) of each variable were compared between groups. Significant differences within groups from pre- to post-intervention were displayed by parameter estimates. This analysis tested the null hypothesis that ∆ parameter was 0. If ∆ parameter had a *P*-value lower than the level of significance, the null hypothesis was rejected, indicating that ∆ parameter was significantly different from 0. Pearson correlations were performed to assess the associations between variables. The level of significance was set at *P* < 0.05. The effect size was denoted as *ƞ* ^2^_p_ (partial eta-squared). All statistical analyses were performed using the software SPSS Statistics 20.0 (SPSS, Chicago, IL, USA).

## Results

*W′* remained unaltered for HIT+VIB18 (*ƞ* ^2^_p_ = 0.014) and HIT+VIB30 (*ƞ* ^2^_p_ = 0.014), while it decreased in the HIT group (*ƞ* ^2^_p_ = 0.196), with a tendency for a group x time interaction relative to the non-significant increase in the VIB30 group (*ƞ* ^2^_p_ = 0.040; Table 1). CMJ *P*_max_ (*ƞ* ^2^_p_ = 0.228), MVT (*ƞ* ^2^_p_ = 0.312), and RFD (*ƞ* ^2^_p_ = 0.179) were decreased in the HIT group and were not altered in the HIT+VIB18 (*ƞ* ^2^_p_ = 0.002, *ƞ* ^2^_p_ < 0.001, *ƞ* ^2^_p_ < 0.001 for CMJ *P*_max_, MVT, RFD), HIT+VIB30 (*ƞ* ^2^_p_ = 0.005, *ƞ* ^2^_p_ = 0.058, *ƞ* ^2^_p_ = 0.033 for CMJ *P*_max_, MVT, RFD), and VIB30 (*ƞ* ^2^_p_ = 0.090, *ƞ* ^2^_p_ = 0.007, *ƞ* ^2^_p_ = 0.003 for CMJ *P*_max_, MVT, RFD) groups (Table 1). In the HIT group, there was a significant decrease in peak velocity during CMJ (pre *vs*. post: 3.01 ± 0.32 *vs*. 2.87 ± 0.18 m∙s^−1^; *P* = 0.007, *ƞ* ^2^_p_ = 0.226), while there was no alteration in peak velocity during CMJ in the other training groups (pre *vs*. post: 2.92 ± 0.27 *vs*. 2.94 ± 0.23 m∙s^−1^, *P* = 0.756, *ƞ* ^2^_p_ = 0.003 for HIT+VIB18; 2.91 ± 0.06 *vs*. 2.92 ± 0.07 m∙s^−1^, *P* = 0.966, *ƞ* ^2^_p_ < 0.001 for HIT+VIB30; 3.00 ± 0.11 *vs*. 2.92 ± 0.13 m∙s^−1^, *P* = 0.106, *ƞ* ^2^_p_ = 0.088 for VIB30).

**Table 1:** Finite work capacity above critical power (*W′*), maximal power during countermovement jumping (CMJ *P*_max_), maximal voluntary torque (MVT), and rate of force development (RFD) during the first 200 ms of contraction pre and post training in the 30 Hz WBV training (VIB30), high-intensity interval training (HIT), HIT with 18 Hz whole-body vibration (WBV; HIT+VIB18), and HIT with 30 Hz WBV (HIT+VIB30) group.

	VIB30 (*n* = 8)			HIT (*n* = 8)			HIT+VIB18 (*n* = 8)			HIT+VIB30 (*n* = 9)		
	Pre	Post	Change (%)	Pre	Post	Change (%)	Pre	Post	Change (%)	Pre	Post	Change (%)
*W'* (kJ)	17.8±6.3	19.0±5.7	7.7±13.9	19.2±3.5	16.4±3.6* ^+^	−14.3±11.8	18.7±5.8	18.1±4.0	0.8±24.3	17.7±6.4	17.1±6.4	−3.4±8.2
CMJ *P*_max_ (kW)	4.55±0.90	4.38±0.82	−3.5±3.9	4.56±0.80	4.26±0.79**	−6.3±6.9	4.50±1.05	4.52±0.83	1.9±9.1	4.51±0.68	4.55±0.74	0.7±4.3
MVT (Nm)	176.2±26.0	178.1±15.4	2.1±10.6	178.7±24.7	163.9±27.4**	−8.6±5.7	185.1±30.4	184.7±35.2	−0.5±3.0	179.0±26.4	173.5±30.3	−4.6±5.8
RFD (Nm∙s^−1^)	1102±279	1116±250	3.1±14.5	1115±158	995±172*	−10.5±11.0	1199±236	1202±218	0.6±5.8	1142±253	1097±235	−2.8±16.8

Values are mean ± SD.

*P < 0.05,

**P < 0.01, significant differences within group pre *vs*. post;

^+^ *P* = 0.067, difference between HIT and WBV group pre *vs*. post.

The proportion of MyHC-1 fibers was not altered pre- to post-training in any of the training groups (Table 2). The proportion of MyHC-2A fibers increased in the HIT, HIT+VIB18, and HIT+VIB30 groups, while the proportion of MyHC-2X fibers decreased significantly in the HIT and HIT+VIB18 group and showed a strong tendency for a decrease in the HIT+VIB30 group. There were no differences pre- to post-training in CSA of MyHC-1 fibers in any of the training groups (*ƞ* ^2^_p_ = 0.012, *ƞ* ^2^_p_ = 0.003, *ƞ* ^2^_p_ = 0.005, *ƞ* ^2^_p_ = 0.001 for the HIT, HIT+VIB18, HIT+VIB30, VIB30 groups; Table 2). CSA of the MyHC-2A fibers decreased significantly in the HIT group (*ƞ* ^2^_p_ = 0.133), and remained unaltered in the three other training groups (*ƞ* ^2^_p_ = 0.004, *ƞ* ^2^_p_ = 0.042, *ƞ* ^2^_p_ = 0.008 for the HIT+VIB18, HIT+VIB30, VIB30 groups; Table 2). There was a significant correlation between ∆*W′* and ∆CSA of the MyHC-2A fibers in the HIT group (*y* = 0.23∙*x*—828.7, *R* ^2^ = 0.596, *P* = 0.025).

**Table 2:** Distribution of the myosin heavy chains (MyHC) types and cross-sectional areas (CSA) pre and post training period in the 30 Hz whole-body vibration training (VIB30), the conventional 4x4 min high-intensity training (HIT), HIT with whole-body vibration at 18 Hz (HIT+VIB18) or 30 Hz (HIT+VIB30) instead of conventional rest intervals, groups.

	VIB30 (*n* = 8)		HIT (*n* = 8)		HIT+VIB18 (*n* = 8)		HIT+VIB30 (*n* = 9)	
	Pre	Post	Pre	Post	Pre	Post	Pre	Post
MyHC-1 (%)	49.7 ± 8.2	51.2 ± 13.3	56.2 ± 16.6	55.5 ± 12.7	48.4 ± 13.7	49.0 ± 13.9	57.2 ± 8.3	53.8 ± 8.7
MyHC-2A (%)	41.7 ± 10.6	43.6 ± 14.6	31.4 ± 9.0	37.8 ± 8.8*	39.6 ± 9.9	45.1 ± 11.2*	36.6 ± 9.3	44.0 ± 7.8**
MyHC-2X (%)	8.6 ± 10.9	5.2 ± 5.5	12.4 ± 11.1	6.7 ± 6.7*	12.0 ± 16.3	5.9 ± 7.8**	6.2 ± 4.4	2.2 ± 2.3^✝^
								
CSA MyHC-1 (μm^2^)	4997 ± 941	4957 ± 1319	4568 ± 908	4439 ± 981	4341 ± 923	4407 ± 1024	4767 ± 625	4694 ± 702
CSA MyHC-2A (μm^2^)	5481 ± 766	5584 ± 1212	5764 ± 1122	5317 ± 798*	5366 ± 1666	5436 ± 1896	5785 ± 974	6010 ± 922

Values are mean ± SD.

*P < 0.05,

**P < 0.01, significant differences within group pre *vs*. post training;

^✝^ *P* = 0.053 pre *vs*. post training.

CP (*ƞ* ^2^_p_ = 0.629, *ƞ* ^2^_p_ = 0.463, *ƞ* ^2^_p_ = 0.456 for HIT, HIT+VIB18, HIT+VIB30), *P*_peak_ (*ƞ* ^2^_p_ = 0.521, *ƞ* ^2^_p_ = 0.634, *ƞ* ^2^_p_ = 0.448 for HIT, HIT+VIB18, HIT+VIB30), and overall capillary-to-fiber ratio (*ƞ* ^2^_p_ = 0.341, *ƞ* ^2^_p_ = 0.553, *ƞ* ^2^_p_ = 0.513 for HIT, HIT+VIB18, and HIT+VIB30) increased in all training groups including HIT without significant differences among these training groups (Table 3). The significant increases in CP and *P*_peak_ in the three groups involving HIT were significantly different from the effects in the VIB30 group. The significant increase in overall capillary-to-fiber ratio in the HIT+VIB groups was significantly different from the non-alteration in the VIB30 group (Table 3). *V̇*O_2peak_ (*ƞ* ^2^_p_ = 0.281, *ƞ* ^2^_p_ = 0.320, *ƞ* ^2^_p_ = 0.260 for HIT, HIT+VIB18, HIT+VIB30), peak cardiac output (*ƞ* ^2^_p_ = 0.181, *ƞ* ^2^_p_ = 0.190, *ƞ* ^2^_p_ = 0.236 for HIT, HIT+VIB18, HIT+VIB30), and peak stroke volume (*ƞ* ^2^_p_ = 0.213, *ƞ* ^2^_p_ = 0.200, *ƞ* ^2^_p_ = 0.305 for HIT, HIT+VIB18, HIT+VIB30) increased in all training groups including HIT without any differences between these three HIT groups (Table 3). Peak heart rate decreased for HIT (*ƞ* ^2^_p_ = 0.150), HIT+VIB18 (*ƞ* ^2^_p_ = 0.132), and HIT+VIB30 (*ƞ* ^2^_p_ = 0.369), without any significant group differences (Table 3).

**Table 3:** Cycling power, overall capillary-to-fiber ratio, and cardiovascular variables pre and post training in the 30 Hz whole-body vibration (WBV) training (VIB30), high-intensity interval training (HIT), HIT with 18 Hz WBV (HIT+VIB18), and HIT with 30 Hz WBV (HIT+VIB30) groups.

	VIB30 (*n* = 8)			HIT (*n* = 8)			HIT+VIB18 (*n* = 8)			HIT+VIB30 (*n* = 9)		
	Pre	Post	Change (%)	Pre	Post	Change (%)	Pre	Post	Change (%)	Pre	Post	Change (%)
CP (W)	237.7±33.4	235.1±36.0	−1.2±2.9	244.7±44.8	278.6±29.1*** ^###^	15.4±10.3	242.3±37.4	266.5±41.9*** ^##^	9.9±2.2	252.8±34.5	275.3±29.8*** ^##^	9.3±5.5
*P*_*peak*_ (W)	331.8±34.3	335.1±39.5	0.9±2.9	354.3±37.2	382.5±29.0*** ^##^	8.4±5.9	340.0±46.9	375.6±46.4*** ^##^	10.7±3.6	357.0±33.1	380.0±32.8*** ^#^	6.6±4.3
												
Overall capillary-to-fiber ratio	1.75±0.23	1.77±0.14	1.1±1.5	1.74±0.28	1.83±0.24***	5.5±5.0	1.66±0.27	1.79±0.26*** ^##^	8.4±5.9	1.83±0.22	1.94±0.25*** ^#^	6.4±3.6
												
˙*V̇*O_2peak_ (l∙min^−1^)	3.82±0.52	3.83±0.50	0.1±1.3	3.83±0.30	4.02±0.29**	5.2±4.7	3.84±0.53	4.05±0.49**	6.0±8.3	3.95±0.39	4.12±0.38**	4.5±1.9
Peak cardiac output (l∙min^−1^)	17.8±4.5	18.0±3.0	3.9±15.8	17.7±3.5	19.2±3.6*	9.2±11.5	16.6±3.1	18.1±3.3*	9.4±3.3	17.8±3.6	19.4±3.2**	8.2±6.2
Peak stroke volume (ml)	100.8±27.0	99.9±15.4	3.9±17.0	92.6±16.0	102.0±17.2**	11.2±13.0	88.3±17.1	97.9±19.0*	10.9±3.5	94.0±20.1	105.6±18.2**	11.5±7.8
Peak heart rate (min^−1^)	180.5±7.2	180.3±6.1	−0.1±3.3	186.3±9.0	182.6±5.2*	−1.9±2.5	188.9±4.2	185.5±3.6*	−1.8±2.0	189.4±4.6	183.2±4.941***	−3.3±1.7

Values are mean ± SD. CP, critical power; *P*_peak_, peak cycling power; *V̇*O_2peak_, peak oxygen uptake.

*P < 0.05,

**P < 0.01,

***P < 0.001 significant differences within group pre *vs*. post;

^#^ *P* < 0.05,

^##^ *P* < 0.05,

^###^ *P* < 0.001 difference between group and VIB30 group pre *vs*. post training.

There were no group differences in average power, average heart rate, and average *V̇*O_2_ during the high-intensity intervals during the 1^st^ and 20^th^ training sessions (Table 4). The increases in average power during the high-intensity intervals between the 1^st^ and 20^th^ training session were not different between the HIT, HIT+VIB18, and HIT+VIB30 groups. Average *V̇*O_2_ during the rest intervals was higher in the HIT compared to the HIT+VIB18 and HIT+VIB30 groups in both training sessions. During the 20^th^ training session, average heart rate during the rest intervals was significantly higher in the HIT than in the HIT+VIB30 group. There were no group differences in individual perception of breathlessness, respiratory exertion, leg exertion, and pain between the HIT, HIT+VIB18, and HIT+VIB30 groups (Table 4).

**Table 4:** Average values for power, heart rate (HR), oxygen uptake (*V̇*O_2_), blood lactate concentration ([La^−^]), and ratings of perceived exertion (PE) during the high-intensity intervals (HI) and rest intervals (RI) during the 1^st^ and 20^th^ training session for the high-intensity interval training (HIT), HIT+VIB18, and HIT+VIB30 groups.

	1^st^ Training			20^th^ Training		
	HIT (*n* = 8)	HIT+VIB18 (*n* = 8)	HIT+VIB30 (*n* = 9)	HIT (*n* = 8)	HIT+VIB18 (*n* = 8)	HIT+VIB30 (*n* = 9)
Power HI (W)	254.2 ± 30.9	247.1 ± 42.1	251.5 ± 30.2	284.9 ± 24.0	279.8 ± 36.9	285.5 ± 22.8
						
HR HI (min^−1^)	172.9 ± 10.0	170.9 ± 5.1	171.3 ± 6.8	171.4 ± 5.9	170.3 ± 5.6	166.4 ± 7.1
HR HI (%peak HR)	92.8 ± 2.0	90.5 ± 2.3	90.4 ± 1.9	93.8 ± 1.6	91.8 ± 1.9	90.8 ± 2.4
HR RI (min^−1^)	164.0 ± 13.0	157.9 ± 9.1	154.0 ± 10.9	163.3 ± 5.9	152.5 ± 9.4	146.6 ± 11.2*
HR RI (%peak HR)	88.0 ± 4.7	83.6 ± 4.9	81.2 ± 3.8	89.4 ± 2.9	82.2 ± 4.6*	79.9 ± 4.1***
						
*V̇*O_2_ HI (l∙min^−1^)	3.29 ± 0.25	3.24 ± 0.48	3.34 ± 0.44	3.65 ± 0.39	3.57 ± 0.33	3.57 ± 0.33
*V̇*O_2_ HI (%*V̇*O_2peak_)	86.1 ± 4.8	84.3 ± 4.0	84.5 ± 7.2	88.1 ± 10.8	88.6 ± 6.6	84.1 ± 6.2
*V̇*O_2_ RI (l∙min^−1^)	2.78 ± 0.16	1.87 ± 0.25***	1.96 ± 0.20***	2.97 ± 0.28	1.86 ± 0.31***	1.89 ± 0.19***
*V̇*O_2_ RI (%*V̇*O_2peak_)	72.9 ± 4.1	47.2 ± 5.4***	49.7 ± 4.3***	71.6 ± 7.4	44.4 ± 5.9***	44.5 ± 4.1***
						
[La^−^] (mmol∙l^−1^)	9.73 ± 3.23	10.60 ± 1.95	11.17 ± 2.06	11.95 ± 2.56	12.75 ± 2.38	11.73 ± 2.40
						
PE breathlessness	3.5 ± 2.2	5.6 ± 3.2	3.8 ± 2.4	6.2 ± 3.3	7.4 ± 3.1	6.4 ± 2.9
PE respiratory exertion	6.0 ± 2.2	7.2 ± 1.9	5.6 ± 2.6	7.8 ± 2.2	8.5 ± 0.9	8.0 ± 1.7
PE leg exertion	7.1 ± 1.9	6.5 ± 0.6	5.9 ± 2.5	7.9 ± 2.0	7.7 ± 2.0	7.3 ± 2.3
PE Pain	4.0 ± 2.5	4.2 ± 3.3	2.8 ± 2.5	7.2 ± 2.1	5.6 ± 4.0	4.0 ± 3.9

Values are mean ± SD.

*P < 0.05,

***P < 0.001, significant differences relative to HIT training group.

## Discussion

Several new findings were obtained with this study. First, the decreases of finite work capacity above CP and anaerobic performance (CMJ *P*_max_, MVT, RFD, and CSAs of MyHC-2A-fibers) after classical HIT could be prevented when the active rest intervals during HIT were replaced with side-alternating WBV. Second, the replacement of active rest intervals during HIT with side-alternating WBV did not interfere with the increases in cycling power (CP and *P*_peak_) and cardiovascular variables (*V̇*O_2peak_, peak cardiac output, and capillary-to-fiber ratio) of the classical HIT protocol. Third, side-alternating WBV intervals had no influence on ratings of perceived exertion during the high-intensity intervals, compared to classical HIT.

The decreases in *W′*, MVT, RFD and CMJ *P*_max_ in the classical HIT group is most likely due to the reduction in CSA of the MyHC-2A fibers. The effect of MyHC-2A fiber atrophy on the decrease in *W′* was supported by the significant correlation between the decrease in fiber CSA and the decrease in *W′* in the HIT group. It is well established that a lower share of MyHC-2 fibers leads to reduced force and power production and *vice versa* [30]. The reduced share of MyHC-2 fibers is augmented by the altered MyHC fiber type distribution. The decreased proportion of MyHC-2X fibers, which is an adaptation to exercise *per se*, leads to a reduced maximal shortening velocity [12]. Thereby, maximal power production was reduced further. Our finding that the CSA of MyHC-2 fibers decreased after HIT is supported by the results of Kohn *et al*. [11], who demonstrated that CSA of MyHC-2 fibers had a tendency to decrease after 6 weeks of HIT in well-trained runners. A decrease in anaerobic performance after HIT is supported by Breil *et al*. [10], who showed that CMJ *P*_max_ is decreased seven days after a HIT shock microcycle and by Faude *et al*. [31], who showed that CMJ height is reduced after HIT in soccer players. However, both research groups explained their results with accumulating fatigue. In light of fact that both studies attained similar results, while viewing the findings of our own study, we presume that these authors focused the explanation of their unexpected results on the factor of fatigue, notwithstanding that there might have been more salient factors present. Our result that anaerobic performance was reduced after cycling HIT, contrasts the findings of Gross *et al*. [32] and Scribbans *et al*. [33], who reported no alterations or even increases, respectively, in anaerobic performance. The apparent discrepancy might be explained by the use of different HIT protocols: HIT with longer high-intensity intervals (*e*.*g*. 4 min, such as in our study) seems to reduce anaerobic performance while HIT with short high-intensity intervals (*e*.*g*. 20 s, such as in the other studies) interspersed with short rest intervals (*e*.*g*. 10 s) may have no effect on anaerobic performance or may even be beneficial in this regard. Decreases in *W′*, MVT, RFD, and CMJ *P*_max_ did not occur when rest intervals during classical HIT (*i*.*e*. including low-intensity cycling active rest intervals) were replaced with side-alternating WBV, indicating that side-alternating WBV plays a very specific role in mediating this effect. This finding is supported by our result that the VIB30 group had a non-significant increase in *W′* of 1.1 kJ that led to a tendency for a group x time interaction with the HIT group.

The efficiency of replacing the active rest intervals during HIT with WBV was proven by the similar training adaptations in cycling power (CP and *P*_peak_), cardiovascular variables (*V̇*O_2peak_, peak cardiac output, peak stroke volume, overall capillary-to-fiber ratio), as well as by MyHC fiber distribution between the HIT+VIB groups and the classical HIT group. Thus, the decreases in anaerobic performance and finite work capacity above CP were prevented, without reduced gains in aerobic performance and capacity. Therefore, both classical HIT and HIT combined with side-alternating WBV improved central (*e*.*g*. peak cardiac output) and peripheral (*e*.*g*. capillary-to-fiber ratio) factors, determining aerobic function. Consequently, we were able to demonstrate that it is feasible to replace cycling during the active rest intervals with WBV without compromising aerobic training adaptations. In contrast, it might be argued that the slightly higher increase in CP in the HIT group compared to the HIT+VIB groups may improve also high-intensity performance and thereby settle the decrease in *W′*. Based on our results, this mechanism might be true for performances longer than 2 min. For example, an athlete with a CP and a *W′* equal to the average post training values of the HIT and HIT+VIB18 groups would be able to sustain 400 W according to the hyperbolic CP model in both cases for 135 s. However, competition-deciding tasks in road cycling or other endurance-oriented sports are clearly shorter (*i*.*e*. sprinting for ∼5–30 s). An athlete would be able to generate a power output of 826 W and 869 W for 30 s based on the average post-training characteristics of the HIT and HIT+VIB18 group, respectively. Hence, during a maximal 30 s sprinting bout, the HIT+VIB group is able to generate ∼5% more power after the training intervention. This calculation is supported by the ∼5–15% higher post-training values in CMJ *P*_max_, RFD, and MVT for the HIT+VIB groups compared to the HIT group, while all groups were starting from similar baseline values.

The above-mentioned training adaptations accompany the similar acute effects during the high-intensity intervals. There were no differences between HIT groups (HIT, HIT+VIB18 and HIT+VIB30) in average power, average heart rate and average *V̇*O_2_. Furthermore, the similar blood lactate concentrations in the three HIT groups point to an equal contribution of anaerobic ATP generation to total energy production in these groups. Thus, our findings lend further credence to the notion that the high-intensity intervals are responsible for the aerobic adaptations, as long as time at or near *V̇*O_2peak_ is not drastically reduced by extended rest intervals or inappropriate intensities of the rest intervals. WBV alone (VIB30), and in combination with HIT (HIT+VIB18 and HIT+VIB30), did neither elicit any changes in the measured aerobic variables, nor improve aerobic adaptations achieved with HIT alone, respectively. Hence, the aerobic adaptations can completely be attributed to the high-intensity intervals. The ratings of perceived exertion also did not differ between the training groups, which indicated that maintaining a half-squat position on a vibration plate, instead of cycling at lower intensities, had neither a beneficial nor a disadvantageous effect on respiratory and leg exertion, nor on pain during the subsequent high-intensity intervals.

Regarding all tested variables, the two vibration frequencies of 18 and 30 Hz did not induce different training adaptations. These results are in line with similar electromyography and acceleration values with vibration frequencies above 15 Hz in the thigh muscles [23]. During WBV, the equal activation of the *M*. *quadriceps*, which is the muscle mainly responsible for force and power production during the exercise tests (knee extension, two-legged jumping, and cycling), led to similar muscular training adaptations. The prevention of decreases in finite work capacity above CP and anaerobic performance due to WBV and the included neuronal adaptation might have been supported by the fatigued state of our participants during WBV. The participants were transferring to the vibration plate after each of the high-intensity intervals. Hence, they were conducting WBV in a fatigued state, and the muscle activation that might thereby have been increased, may have led to neuronal adaptations. This mechanism is in line with a recent study investigating the effect of fatigue on vertical WBV, showing that maximal electromyography activity was significantly higher after fatiguing exercise [34]. Summarized, when HIT is combined with side-alternating WBV, vibration frequencies between 18 and 30 Hz will be equally effective in preventing decreases in anaerobic performance and capacity. Under these circumstances, the subjectively perceived exertion might turn the balance to 18 Hz, which is usually better tolerated by participants.

The results of the present study might have a direct practical impact. The prevention of decreases in anaerobic performance and finite work capacity above CP might be critical indicators in most endurance-oriented sports. In most endurance disciplines, the decision between victory and defeat is based on fatigue resistance in conjunction with anaerobic performance and capacity (*e*.*g*. sprinting). The combination of aerobic HIT with side-alternating WBV allows an increase in aerobic function, without negatively affecting finite work capacity above CP and anaerobic performance. A similar effect might be possible by simply adding resistance training to the training routine since it was shown that resistance training increases *W′* without affecting CP [35]. However, HIT and resistance training cannot be performed within one training session without an inevitable loss of intensity for the training modality performed as the second. Hence, HIT and resistance training probably would have to be performed during separate training sessions, which in turn would cause an increase in time commitment. As we showed in the present study, significant results could be achieved without increasing training time simply by using the HIT rest intervals more efficiently. Hence, time efficiency and overall effectiveness of training could be markedly increased, obviating the need to separately perform resistance training (notably if the sole aim of resistance training is to prevent the decrease in finite work capacity above CP).

The present study had at least three limitations. First, as the employed training devices varied as a function of training group, it was not possible to blind the participants and supervisors to group allocation during the training intervention. Hence, it cannot be excluded that the mere awareness about the existence of different training groups may have influenced the participants' training behavior, and, consequently, the study results. Nevertheless, all participants expected an increase in physical performance ability in response to their training interventions, and the participants, as well as the training supervisors, were not aware of the exact scientific objectives and the working hypotheses. Based on these two criteria, we can presume that knowledge about the group assignment influenced neither training behavior nor performance of participants, and therefore, did not make an impact on the study outcomes. Second, we would like to point out that only cycling HIT was investigated in this study. It might be possible that different training effects occur with other exercise modalities, *e*.*g*. running. Third, the participants were recreationally active but not athletes. There might be distinct training adaptations in athletes. However, as we assume that the decrease in CSA of MyHC-2A fibers is related to energy stress and energy stress is even higher in athletes, we expect similar decreases in CSA. Nonetheless, it remains to be investigated if WBV prevents possible decreases in CSA also in athletes.

## Conclusions

In conclusion, reductions in *W′*, CSA of MyHC-2A fibers, MVT, RFD and CMJ *P*_max_ that were characteristic for the classical 4x4 min HIT protocol were prevented by the addition of side-alternating WBV during the rest intervals. The replacement of low-intensity cycling during active rest intervals during a 4x4 min HIT with WBV led to similar adaptations in cycling power, and cardiovascular variables. To prevent MyHC-2A fiber atrophy as well as to prevent decreases in *W′*, CSA of MyHC-2A fibers, MVT, RFD and CMJ *P*_max_ without additional time commitment, athletes could benefit by complementing aerobic HIT with side-alternating WBV, since it was observed in recreationally active participants. This is of particular importance for athletes engaged in most endurance-oriented sports that involve anaerobic tasks (*e*.*g*. sprinting).
